# Role of Ultrasonography and Color Doppler in the Assessment of High-Risk Pregnancies and Their Accuracy in Predicting Fetal Outcome

**DOI:** 10.7759/cureus.39017

**Published:** 2023-05-15

**Authors:** R. Mahima Kale, Rajeswari G Tirupathi, S. R. Sheela

**Affiliations:** 1 Department of Radiodiagnosis, Sri Devaraj Urs Academy of Higher Education and Research, Kolar, IND; 2 Department of Obstetrics and Gynecology, Sri Devaraj Urs Academy of Higher Education and Research, Kolar, IND

**Keywords:** color doppler, mca, ua, fetal outcome, high-risk pregnancies

## Abstract

Introduction

Various methods are employed to evaluate the well-being of the fetus in high-risk pregnancies which consists of a biophysical profile (BPP), a non-stress test (NST), and daily fetal movements. Detection of aberrant blood flow in fetoplacental beds has been revolutionized by recent developments in ultrasound technology, such as color Doppler flow velocimetry. The cornerstone of maternal and fetal care is lowering maternal and perinatal mortality and morbidity is antepartum fetal surveillance. Doppler ultrasound is a non-invasive way of obtaining a qualitative and quantitative evaluation of maternal and fetal circulation and is utilized to investigate complications like fetal growth restriction (FGR) and fetal distress. Thus, it is useful in making distinctions between fetuses that are truly growth restricted and small for gestational age and healthy fetuses. The aim of the current study was to determine the role of Doppler indices in high-risk pregnancies and their accuracy in predicting fetal outcomes.

Material and methods

This prospective cohort study included 90 high-risk pregnancies in the III trimester (after 28 weeks of gestation) on whom ultrasonography and Doppler were performed. Ultrasonography was performed using PHILIPS EPIQ 5, a curvilinear probe of frequency 2-5MHz. Gestational age was determined with a biparietal diameter (BPD), head circumference (HC), abdominal circumference (AC), and femoral length (FL). Placental grading and position were noted. Estimated fetal weight and the amniotic fluid index were calculated. BPP scoring was done. Doppler study was conducted and the findings of Doppler indices that is pulsatility index (PI) and resistive index (RI) of the middle cerebral artery (MCA), umbilical artery (UA), and uterine artery (UTA), and cerebroplacental (CP) ratio in these high-risk pregnancies were documented and compared with standard. The flow patterns in MCA, UA, and UTA were also assessed. These findings were correlated with fetal outcomes.

Results

Among 90 cases, the common high-risk factor in pregnancy was preeclampsia without severe features (30%). Growth lag was present in 43 (47.8%) participants. Among the study population, HC/AC ratio was increased in 19 (21.1%) participants which indicates asymmetrical intrauterine growth restriction. Adverse fetal outcomes were seen in 59 (65.6%) of the subjects. CP ratio and UA PI had better sensitivity (83.05% and 79.66%, respectively) and positive predictive value (PPV) (87.50% and 90.38%, respectively) in identifying the adverse fetal outcomes. Diagnostic accuracy of CP ratio and UA PI (Accuracy=81.11%) was highest in predicting adverse outcomes than all the other parameters.

Conclusion

CP ratio and UA PI had better sensitivity, PPV, and diagnostic accuracy in identifying adverse fetal outcomes than other parameters. The study's findings support that the use of color Doppler imaging in high-risk pregnancies will help in the early identification of adverse fetal outcomes and aid in early intervention. This study is non-invasive, simple, safe, and reproducible. This study can also be performed bedside in high risk and unstable patients. This study is required to accurately assess fetal well-being in all high-risk pregnancies in order to improve fetal outcomes and to incorporate this procedure as a part of the protocol for the assessment of fetal well-being in these patients.

## Introduction

The incidence of preeclampsia is 8%-10% in pregnant women, according to the National Health Portal of India. In India, the research found that the frequency of hypertensive disorders during pregnancy was 7.8%, with preeclampsia (PE) occurring in 5.4% of the population [1]. Various methods are utilized to evaluate the well-being of fetuses in high-risk pregnancies which include non-stress tests (NST), biophysical profile (BPP), and daily fetal movements. All the above tests are less desirable since they lack a high level of positive predictive value (PPV), sensitivity, and specificity.

The identification of abnormal blood flow in fetoplacental beds has been revolutionized by recent advances in ultrasound (USG) such as Color Doppler flow velocimetry. Early detection of these abnormalities is helpful in determining the optimal time for delivery and early diagnosis of intra-uterine growth restriction (IUGR) which reduces fetal mortality and morbidity.

This technique demonstrates blood flow in uterine arteries (UTAs), umbilical artery (UA), and middle cerebral artery (MCA). This is a non-invasive technique to study uteroplacental and fetoplacental circulations. It is simple, safe, and reproducible. Fetal hypoxia can be assessed with the abnormal wave patterns obtained from these vessels. This method makes it easier to delineate small intracranial vessels resulting in quicker and more accurate examinations. The aim of the current study was to determine the role of Doppler indices in high-risk pregnancies and their accuracy in predicting fetal outcomes.

Fetal growth restriction (FGR) and fetal distress that results due to fetal hypoxemia or asphyxia can be investigated using Doppler velocimetry of uteroplacental and fetoplacental circulations [2]. Thus, it is useful in distinguishing a healthy fetus from a fetus that is truly growth restricted from small for gestational age. Therefore, the current study is important for the accurate assessment of the well-being of fetuses in high-risk pregnancies in order to improve fetal outcomes.

## Materials and methods

This is a prospective cohort study conducted at a tertiary hospital, R. L. Jalappa Hospital and Research Center in Kolar, Karnataka, India, over a period of 18 months (January 2021 and July 2022). All singleton pregnancies beyond 28 weeks of gestation (third trimester) deemed by investigators to be at high risks like preeclampsia, eclampsia, and gestational diabetes mellitus (GDM) were included in the study. Pregnancies with any congenital anomalies and multiple pregnancies were excluded from the study.

The sample size was calculated as 87 using master software version 2.0. The study was approved by the institutional ethics committee of Sri Devaraj Urs Medical College, Kolar, Karnataka, India (approval number: SDUMC/KLR/IEC/603/2020-21). Written informed consent was obtained from all the study participants and only those participants willing to sign the informed consent were included in the study. The risks and benefits involved in the study and the voluntary nature of participation were explained before obtaining consent. The confidentiality of the study was maintained.

A detailed history was taken from all the patients meeting the inclusion criteria and referred to the Department of Radiodiagnosis. Patients were subjected to ultrasonography examination and a Doppler study. The equipment used was PHILIPS EPIQ 5G system with a pulsed wave, continuous wave, and HPR Doppler with dual sector transducer. The scan was performed using 2D real-time USG with a C5-1 MHz convex sector transducer. Gestational age was determined with a biparietal diameter (BPD), head circumference (HC), abdominal circumference (AC), and femoral length (FL). Placental grading and position were noted. Estimated fetal weight and the amniotic fluid index were calculated. BPP scoring was done. Doppler study was conducted and the findings of Doppler indices like pulsatility index (PI) and resistive index (RI) of the MCA, UA, and UTA, and cerebroplacental (CP) ratio in these high-risk pregnancies was documented and compared with standard. The flow patterns in MCA, UA, and UTA were also assessed. This was correlated with fetal outcomes. The data were entered into a Microsoft Excel sheet and the SPSS 22 version (IBM Corp., Armonk, NY) of the software was used to analyze the data. The sensitivity, specificity, PPV, NPV, and diagnostic accuracy of each Doppler indices in predicting the adverse fetal outcome are calculated.

## Results

A total of 90 subjects were included in the study, of which the maximum number of cases were in the age group between 21 and 25 years (39 participants; 43.3%), followed by 26-30 years (26 participants; 28.9%), less than 20 years (15 participants; 16.7%) and least number of cases were seen beyond 30 years (10 participants; 11.1%). The age distribution based on maternal age is shown in Table 1. The mean age group of subjects was 25.4 years and the age group ranged between 18 and 37 years. A total of 49 (54.4%) participants were multigravida and formed the majority of the study population, while 41 (45.6%) patients were primigravida.

**Table 1:** Distribution of subjects based on maternal age.

Age (Years)	Frequency	Percentage
<20	15	16.7
21-25	39	43.3
26-30	26	28.9
>30	10	11.1
Total	90	100.0

Among the study population, the most common high-risk pregnancy was PE without severe features (27 participants; 30.0%), followed by eclampsia (24 participants; 26.7%), gestational diabetes (21 participants; 23.3%) and PE with severe features (18 participants; 20.0%) as shown in Table 2.

**Table 2:** Distribution of subjects based on high-risk pregnancies.

High-risk pregnancies	Frequency	Percentage
Preeclampsia without severe features	27	30.0
Preeclampsia with severe features	18	20.0
Eclampsia	24	26.7
GDM	21	23.3
Total	90	100.0

Oligohydramnios was seen in 37 (41.1%) participants and six (6.7%) participants had polyhydramnios. Growth lag was present in 43 (47.8%) participants while, HC/AC ratio was increased in 19 (21.1%) participants which indicates that these participants had asymmetrical IUGR.

Abnormal (below < 5th percentile) MCA PI was seen in 42 (46.7%) subjects and abnormal (below < 5th percentile) MCA RI was seen in 28 (68.9%) subjects. Abnormal (above > 95th percentile) UA PI and UTA PI was seen in 56 (62.2%) and 47 (52.2%) subjects, respectively, and abnormal (above > 95th percentile) UA RI 43 (68.9%) and 45 (50%) subjects, respectively. The distribution of various Doppler indices is shown in Table 3.

**Table 3:** Distribution of subjects based on resistive index (RI) and pulsatility index (PI) of middle cerebral artery (MCA), umbilical artery (UA) and uterine artery (UTA).

	MCA PI		MCA RI		UA PI		UA RI		Mean UTA PI		Mean UTA RI	
	Frequency	Percentage	Frequency	Percentage	Frequency	Percentage	Frequency	Percentage	Frequency	Percentage	Frequency	Percentage
Normal	48	53.3	62	68.9	34	37.8	47	52.2	43	47.8	45	50.0
Abnormal	42	46.7	28	31.1	56	62.2	43	47.8	47	52.2	45	50.0
Total	90	100	90	100	90	100	90	100	90	100	90	100

**Figure 1:**
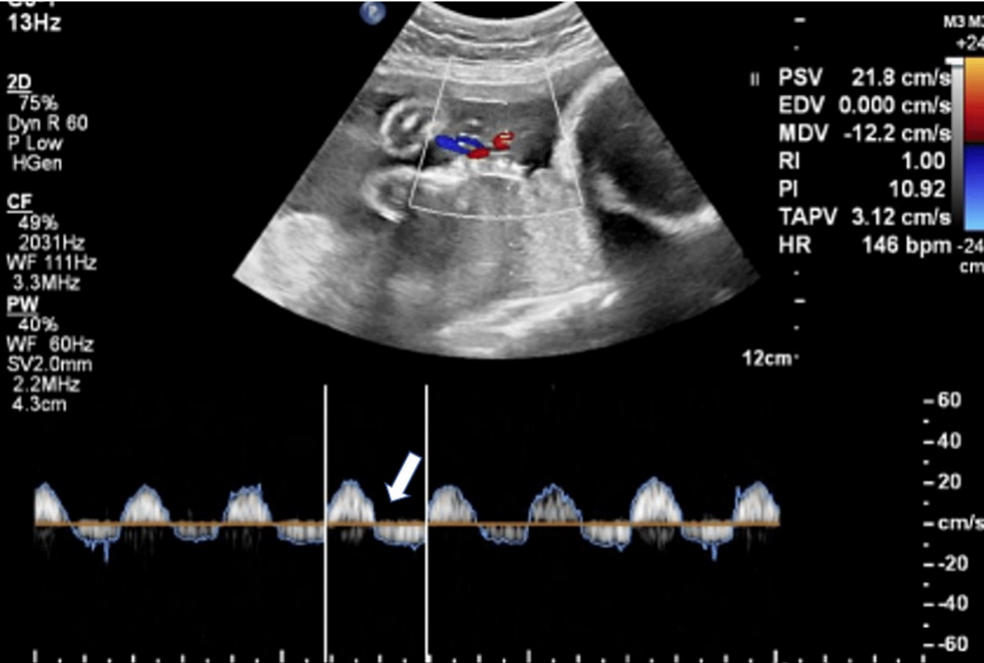
Reversal of end diastolic flow (REDF) in umbilical artery. Spectral Doppler of umbilical artery (UA) show REDF (white arrow) with raised UA pulsatility index (PI) in 22 years old women with preeclampsia without severe features.

**Figure 2:**
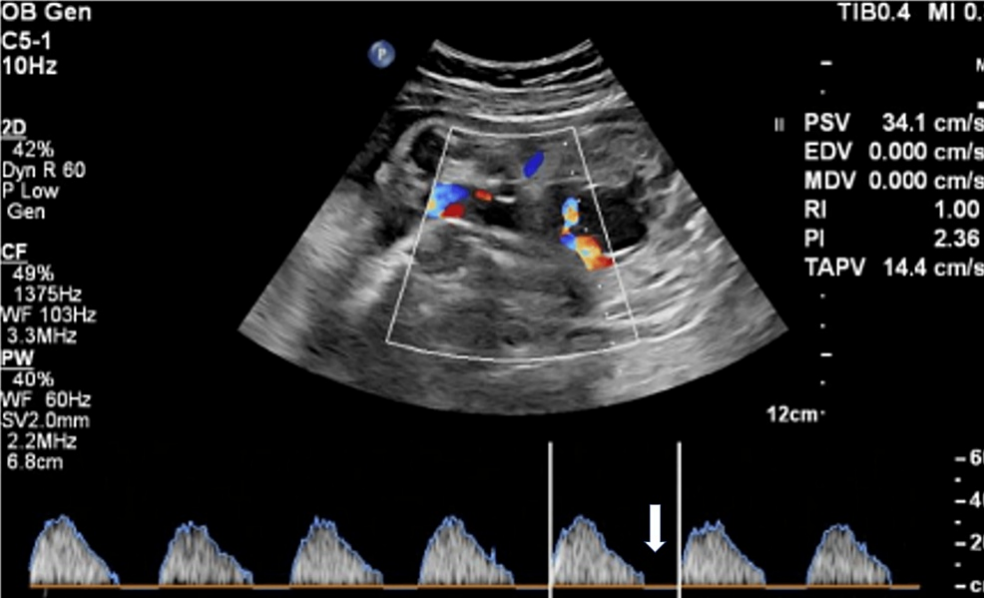
Absent end diastolic flow (AEDF) in umbilical artery. Spectral Doppler of umbilical artery (UA) showing AEDF (white arrow) with raised UA pulsatility index (PI) in 25 years old women with high-risk pregnancy having preeclampsia with severe features.

In our study, it was found that, 40 (44.4%) subjects had normal UA flow, 17 (18.9%) subjects had reduced flow in UA, 15 (16.7%) subjects had absent end diastolic flow (AEDF) and 18 (20%) subjects had reversal of end diastolic flow (REDF) in UA. Distribution of subjects according to UA flow patterns in various high-risk pregnancies is shown in Table 4. Figures 1, 2 show abnormal UA flow patterns.

**Table 4:** Distribution of subjects according to umbilical artery flow patterns in various high-risk pregnancies.

UA flow patterns	Preeclampsia without severe features	Preeclampsia with severe features	Eclampsia	GDM
	Frequency	Frequency	Frequency	Frequency
Absent	5	3	7	0
Normal	9	5	5	21
Reduced	6	4	7	0
Reversal	7	6	5	0

**Figure 3:**
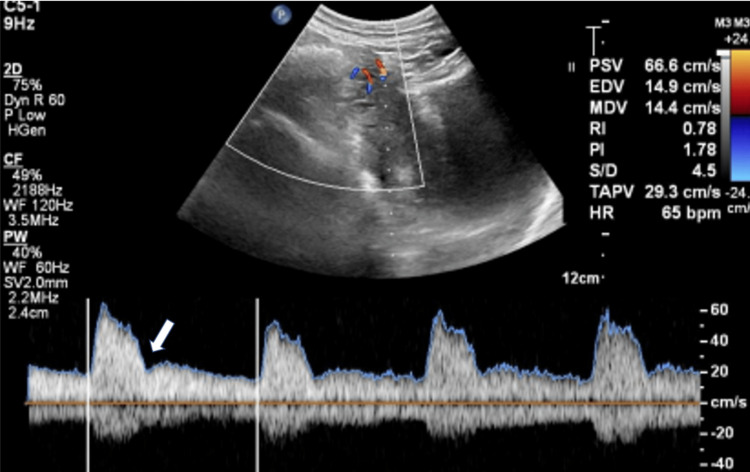
Early diastolic notching in uterine artery. Spectral Doppler of right uterine artery in 26 years old women with preeclampsia without severe features showing early diastolic notching with raised uterine artery pulsatility index (UTA PI).

Normal UTA flow was seen in 38 (42.2%) subjects and 52 (57.9%) subjects had early diastolic notching in UTAs. The distribution of early diastolic notching in UTAs in various high-risk pregnancies is described in Table 5. Early diastolic notching is shown in Figure 3.

**Table 5:** Distribution of subjects based on flow in uterine artery in high-risk pregnancies.

High-risk conditions	Early diastolic notching in uterine artery	
	Unilateral	Bilateral
Preeclampsia without severe features	10	12
Preeclampsia with severe features	4	8
Eclampsia	7	6
GDM	3	2

Reversal of CP ratio was seen in 52 (57.8%) subjects. Distribution of subjects based on CP ratio in all high-risk pregnancies is shown in Table 6.

**Table 6:** Distribution of subjects according to CP ratio.

	Preeclampsia without severe features	Preeclampsia with severe features	Eclampsia	GDM
	N	N	N	N
Normal	6	5	6	21
Reversal	21	13	18	0

Fetoplacental insufficiency was seen in 57 (63.3%) cases and P-value < 0.001, there was a statistically significant difference found between fetoplacental insufficiency and outcome. Uteroplacental insufficiency was seen in 62 (68.9%) cases and P-value 0.016, there was a statistically significant difference found between uteroplacental insufficiency and outcome.

Among the study population, 31 subjects underwent normal vaginal delivery (NVD) in 31 (34.4%) and 59 (65.5%) underwent lower segment cesarean section. Adverse fetal outcomes were seen in 59 (65.6%) of the subjects. The distribution of subjects according to maternal complication and outcome is shown in Table 7 and PE with severe features had the highest adverse outcomes. P-value was <0.001, there was a statistically significant difference found between complication and outcome.

**Table 7:** Distribution of subjects according to complication and outcome.

	Uneventful		Adverse event	
	N	%	N	%
Preeclampsia without severe features	8	29.6	19	70.3
Preeclampsia with severe features	4	22.2	14	77.7
Eclampsia	5	20.8	19	79.2
GDM	14	66.7	7	33.3

Sensitivity, specificity, NPV, PPV and accuracy for MCA PI, MCA RI, UA PI UA RI, UTA PI, UTA RI and CP ratio in predicting adverse fetal outcome is shown in Table 8.

**Table 8:** Sensitivity, specificity, NPV, PPV and accuracy for MCA PI, MCA RI, UA PI UA RI, UTA PI, UTA RI, and CP ratio in predicting adverse fetal outcome.

Statistic	Sensitivity	Specificity	Positive Predictive Value	Negative Predictive Value	Accuracy
MCA PI	66.10%	90.32%	92.86%	58.33%	74.44%
MCA RI	40.68%	87.10%	85.71%	43.55%	56.67%
UA PI	83.05%	77.42%	87.50%	70.59%	81.11%
UA RI	59.32%	74.19%	81.40%	48.94%	64.44%
UTA PI	62.71%	67.74%	78.72%	48.84%	64.44%
UTA RI	55.93%	61.29%	73.33%	42.22%	57.78%
CP Ratio	79.66%	83.87%	90.38%	68.42%	81.11%

## Discussion

Studies using perinatal Doppler velocimetry are able to identify fetuses who are at risk of having adverse outcomes, allowing for prompt intervention. It is challenging to analyze each parameter independently with different research because the definition of adverse fetal outcomes is not fixed. Since various clinical factors and the Doppler results were taken into consideration for patient management in the current study, it is possible that fetal outcomes will differ from that of other studies. We studied the role of color Doppler and USG in high-risk pregnancies. Doppler finding’s predictive value in various high-risk pregnancies was studied and its significance in the management of fetal outcomes was established.

In the current study, out of 90 (100%) patients, most of the patients were in the age group 21-25 years (39/43.3%). In the current study, the mean age was 25.4 years, ranging between 18 and 37 years in the study population. Age ranged from 19 to 35 years and the mean was calculated as 26.73 years in a study conducted by Gaikwad et al. which is similar to our study [3]. A similar finding was found in a study by Kavitha et al. where most of the patients were in the age group 26-30 years (n=80) followed by 20-25 years (n=60) [4].

According to complications, out of 90 (100%) majority of the patients had PE without severe features (27/30%), followed by eclampsia (24/26.7%), GDM (21/23.3%), PE with severe features (18/20%). A statistically significant difference was found between complication and outcome with a p-value of <0.001. This is similar to the study of Aparna et al. about 90% of patients at risk had PE and 6% had gestational diabetes [5]. In our study, out of the 90 patients, 54.4% were multigravidas and the rest were primigravidas.

In our study, most of the patients (55/61.1%) were in >36 weeks of the gestation period, followed by 25 (27.8%) of the patients who were in 32-36 weeks and 10 (11.1%) were in 28-32 weeks. Aparna et al. had patients with a gestational age of 28 to 37 weeks [5].

Among the study population, 47 (52.2%) participants had normal amniotic fluid volume, 37 (41.1%) participants had oligohydramnios, and six (6.7%) participants had polyhydramnios. Oligohydramnios was seen in 20% of the patients in a study conducted by Kavitha et al. [4].

Among the total study population, growth lag was present in 43 (47.8%) participants. According to the fetal HC/AC ratio, out of 90 (100%) patients, 71 (78.9%) of them were found to be in the normal range and about 19 (21,2%) of them had increased HC/AC ratio which indicated asymmetrical IUGR in fetuses.

Out of 90 (100%) patients, 47 (52.2%) participants had normal amniotic fluid volume, 37 (41.1%) participants had oligohydramnios, and 6 (6.7%) participants had polyhydramnios. Out of 90 (100%) patients, most of them (62/53.3%) had normal MCA-RI and (41/46.7%) normal MCA-PI. Twenty eight (31.1%) of the patients had abnormal MCA-RI and 42 (46.7%) had abnormal MCA-PI.

In our study, out of 90 (100%) patients, most of them (47/53.2%) had normal UA-RI whereas most of the patients 56 (62.2%) had abnormal UA-PI. In the present study, 40 (44.4%) subjects had normal UA flow pattern, 17 (18.9%) subjects had reduced flow in the UA, 15 (16.7%) subjects had AEDF, and 18 (20%) subjects had a REDF in the UA. Kumbar et al. [6] evaluated the role of Doppler parameters in IUGR babies and found that 50% of perinatal deaths were seen in cases showing absent diastolic flow and 100% in cases having reverse end diastolic flow. In Gaikwad et al., 50% mortality in cases of REDF and 33.33% mortality was seen in cases with AEDF [3].

In our study, out of 90 (100%) patients, most of them (45/50.0%) had normal UTA-RI whereas most of the patients - 47 (52.2%) of the patients had abnormal UTA-PI. Among the study population, 38 (42.2%) subjects had normal UTA flow, and 52 (57.9%) subjects had early diastolic notching in UTAs. In Kavitha et al. [4] IUD was seen in 67% of fetuses with absent or reversal of diastolic flow. Ochi et al. stated that elevated PI and the presence of a diastolic notch in the UTA flow velocity are indicators of increased uterine arterial resistance and impaired uterine circulation [7].

In the present study, out of 90 (100%) patients, 57 (63.3%) were found to have fetoplacental insufficiency and 62/68.9% were found to have uteroplacental insufficiency. The P-value is <0.001, which showed a statistically significant difference between fetoplacental insufficiency and outcome. P-value 0.016, there was a statistically significant difference found between uteroplacental insufficiency and outcome.

Of the majority of patients out of 90 (100%), about 59 (65.6%) had LSCS mode of delivery, and the remaining (31/34.4%) of the patients had NVD. This is in accordance with the study of Aparna et al., more than 90% of women delivered by cesarean section [5].

Out of 90 (100%) patients, about 59 (65.6%) had adverse event outcomes and 31 (34.4%) had an uneventful outcome. This is similar to the study of Kavitha et al. out of these 15, 14 cases had adverse perinatal outcomes [4]. Gaikwad et al. found adverse perinatal outcomes in 63 (50.4%) patients [3]. Out of 59 (65.6%) adverse event outcome patients, most of the patients (20/74.1%) had PE without severe features followed by eclampsia (19/79.25%), PE with severe features (13/72.2%) and GDM (7/33/3%). A statistically significant difference was found between complication and outcome with a p-value of <0.001. Among 90 (100%) of the patients, the majority of the babies (43/47.8%) had NICU admission, about 32 (43.2%) babies had low birth weight (LBW) and 27 (36.5%) had low APGAR scores. In the Gaikwad study out of a total of 125 babies, 80.8% babies had LBW (< 2.5 kg) [3]. NICU admission was in 88.8% of babies for various reasons like LBW, asphyxia, prematurity, etc.

In the present study, the sensitivity, specificity, NPV, PPV, and diagnostic accuracy for MCA RI in predicting adverse outcomes were 40.68%, 87.10%, 43.55, 85.71%, and 56.67%, respectively. Gaikwad et al. also studied the fetal MCA through Doppler studies; MCA RI was found to be abnormal in 25.6% of patients. It was discovered that MCA RI had the highest specificity of 100% for predicting unfavorable outcomes. Additionally, MCA RI was found to have a PPV of 100% for predicting unfavorable outcomes [3].

Our study found that the sensitivity, specificity, NPV, PPV, and accuracy of UA RI in predicting adverse outcomes were 59.32%, 74.19%, 48.94%, 81.40%, and 64.44%, respectively. In Gaikwad et al.'s study, the UA RI had a higher specificity and PPV of 91.94% and 81.48% in predicting adverse perinatal outcomes. In Gaikwad et al., the correlation between UA RI and pregnancy outcome was found statistically significant (p<0.05) [3]. Another study conducted by Lakshmi et al. [8] showed that the UA RI had a sensitivity of 84.9%, specificity of 72.3%, PPV OF 77.5%, NPV OF 89%, and diagnostic accuracy of 79%, and Lakhkar et al. [9] showed a sensitivity of 44.4% and specificity of 81.8% as described in Table 9.

**Table 9:** Comparison of UA RI ratio in predicting adverse outcome. UA RI - umbilical artery resistive index

Study	Sensitivity	Specificity	PPV	NPV	Diagnostic accuracy
Lakshmi et al^8^	84.9%	72.3%	77.5%	89%	79%
Lakhkar et al^9^	44.4%	81.8%	80%	47.3%	-
Gaikwad et al^3^	34.92%	91.94%	81.48%	58.16%	63.20%
Present study	59.32%	74.19%	48.94%	81.40%	64.44%

The sensitivity, specificity, NPV, PPV, and accuracy for UTA RI in predicting adverse outcome was 55.93%, 61.29%, 73.33%, 42.22%, and 57.78%, respectively. 

Our study showed that the Sensitivity, Specificity, NPV, PPV, and Accuracy of MCA PI in predicting adverse outcomes were 66.10%, 90.32%, 92.86%, 58.33%, and 74.44%. Gaikwad et al. [3] revealed MCA PI had the highest specificity of 98.39% in predicting adverse perinatal outcomes. The correlation between MCA PI and pregnancy outcome was statistically significant (p<0.05). 

The PPV of MCA PI in the present study is 92.31% in predicting unfavorable outcomes. The sensitivity and specificity are comparable with studies of Yash et al., Netam et al., Bano et al., Khanduri et al. and Fong et al. [10-14] as described in Table 10.

**Table 10:** MCA PI in predicting adverse outcomes. MCA PI - middle cerebral artery pulsatility index

Study	Sensitivity	Specificity	PPV	NPV	Diagnostic accuracy
Yash et al^10^	76%	78%	-	-	52%
Lakshmi et al^8^	62,2%	78.7%	76.7%	64,9%	63%
Kumbar et al^6^	78.9%	68.4%	65.2%	76.4%	70%
Netam et al^11^	47.06%	81.81%	57.14%	75%	70%
Bano et al^12^	8.9%	100%	100%	-	-
Khanduri et al^13^	35.7%	92.6%	91.8%	38.2%	-
Fong et al^14^	72.4%	58.1%	37.7%	85.7%	-
Gaikwad et al^3^	19.5%	98.39%	92.31%	54.31%	58.40%
Present study	66.10%	90.32%	58.33%	92.86%	74.44%

We had the sensitivity, specificity, NPV, PPV, and Accuracy for UA PI in predicting adverse outcomes was 83.05%, 77.42%, 87.50%, 70.59%, and 81.11%, respectively. This is similar to the study of Gaikwad where UA RI, and PI had high specificity of 91.94% and 93.55%, respectively, in identifying unfavorable perinatal outcomes [3]. The sensitivity and specificity are comparable with studies of Lakshmi et al., Khanduri et al., and Yoon et al. [8,13,15] as shown in Table 11. The sensitivity, specificity, NPV, PPV, and accuracy of UTA PI in predicting adverse outcome was 62.71%, 67.74%, 78.72%, 48.84%, and 64.44%, respectively. 

**Table 11:** UA PI in predicting adverse outcomes in different studies UA PI - umbilical artery pulsatility index

Study	Sensitivity	Specificity	PPV	NPV	Diagnostic accuracy
Lakshmi et al^8^	86.7%	63%	73%	85.71%	76%
Lakhkar et al^9^	50%	59%	66.6%	41%	-
Kumbar et al^6^	89%	85.7%	85%	90%	87.5%
Khanduri et al^13 ^	73.8%	75.9%	87.7%	55.4%	75%
Bano et al^12^	46.7%	93%	87%	63%	70%
Yoon et al^15^	89%	86%	86%	89%	-
Gaikwad et al^3^	38.10%	75.9%	85.71%	59.79%	65.60%
Present study	83.05%	77.42%	81.11%	70.59%	81.11%

CP ratio is an important predictor of adverse pregnancy outcomes. It is calculated by dividing the Doppler PI of the MCA by the UA PI. 

CP ratio = MCA PI/UA PI. 

The CP ratio is considered abnormal if the ratio is <1. The index will reflect a mild increase in placental resistance with mild reductions in fetal brain vascular resistance. An abnormal CP ratio reflects the redistribution of cardiac output to the cerebral circulation and has been associated with intrapartum fetal distress. 

In the present study, out of 90 (100%) patients, the majority of patients (52/57.8%) had reversed fetal CP ratio. A sensitivity of 79.66%, specificity of 83.87%, NPV of 68.42%, PPV of 90.38% and accuracy of 81.11% for CP ratio in predicting adverse outcome was observed in the present study. In a similar study by Lakshmi et al., it was found that the CP ratio has 90% sensitivity and 94% PPV [8]. CP ratio has the highest specificity and PPV of 90.32% and 83.33% respectively in identifying adverse perinatal outcomes as seen in Gaikwad et al. study [3].

In 2010, Bansal et al. did a study on the role of pan vessel Doppler studies in high-risk pregnancies. Their conclusion was similar to our study, women with aberrant Doppler indices had higher rates of LSCS (78%), low Apgar scores, and LBW and NICU admissions (36%) [16]. In 2010, Urmila and Beena did a study on triple vessel wave patterns in high-risk and normal pregnancies. They came to the same conclusion as ours; in the study group, there was an increased incidence of LSCS and NICU admissions as compared to the control group [17].

In a 2016 study on color Doppler ultrasonography in high-risk pregnancies, Amin et al. found that perinatal mortality and morbidity were 41.3% and 23.9%, respectively, among 46 pregnancies with abnormal Doppler waveforms, in comparison with patients having normal Doppler waveforms, had 3.7% (mortality) and 11.1% (morbidity) [18].

In a study conducted by Kirkinen et al., it was discovered that Doppler investigations had advanced significantly and were now acknowledged as a crucial examination to forecast heart failure in hypoxic fetuses. In 83 low-risk and 84 high-risk pregnancies, the pulsed Doppler method was utilized to record the blood flow velocity waveforms from fetal intracranial arteries. In typical cases, the waveform's RI decreased as the pregnancy progressed, and these arteries continued forward flow. A low RI had a 57% sensitivity and 94% specificity for predicting the birth of an infant that was small for dates and/or the subsequent development of a cardiotocographic abnormality [19].

## Conclusions

CP ratio and UA PI had better sensitivity and PPV in identifying unfavorable outcomes. Diagnostic accuracy of CP ratio and UA PI was highest in predicting adverse outcomes. The study's findings support that the use of color Doppler imaging in high-risk pregnancies will help in the early identification of adverse fetal outcomes and aid in early intervention. This is a non-invasive technique to study uteroplacental and fetoplacental circulation. This study is simple, safe, and reproducible. This study can also be performed bedside in high risk and unstable patients. This study is required to accurately assess fetal well-being in all high-risk pregnancies in order to improve fetal outcomes and to incorporate this procedure as a part of the protocol for the assessment of fetal well-being in these patients.
